# Passive smoking may be associated with bleeding of cerebral arteriovenous malformation in non-smoking women: a retrospective analysis

**DOI:** 10.1590/0004-282X-ANP-2021-0216

**Published:** 2022-08-08

**Authors:** Jiao Wang, Shuai Zhang

**Affiliations:** 1 Beijing Jingmei Group General Hospital, Department of Emergency, Beijing, China.; 2 Beijing Jingmei Group General Hospital, Department of Neurosurgery, Beijing, China.

**Keywords:** Tobacco Smoke Pollution, Hemorrhage, Intracranial Arteriovenous Malformations, Risk, Poluição por fumaça de tabaco, hemorragia, malformações arteriovenosas intracranianas, risco

## Abstract

**Background:**

Smoking has been considered to be a risk factor for cardiovascular disease, cancer, depression and other diseases in previous reports, and active smoking is considered to be a risk factor for hemorrhagic stroke. In addition, a retrospective study showed that male smokers were at increased risk of bleeding from arteriovenous malformation (AVM), compared with non-smokers. However, the effect of passive smoking on rupturing of cerebral AVM in non-smoking women has not been addressed.

**Objective:**

This study aimed to assess the impact of tobacco exposure on AVM bleeding risk in non-smoking women.

**Methods:**

A total of 393 non-smoking women diagnosed with AVM were included. They were divided into a bleeding group (205 women) and a non-bleeding group (188 women). We conducted univariate and multivariate analysis on these two groups. In univariate analysis, risk factors that might be related to AVM bleeding were analyzed. In multivariate analysis, the relationship between passive smoking and AVM rupture was analyzed by correcting confounding factors.

**Results:**

Multivariate analysis showed that the proportion of passive smoking was statistically different between the bleeding group and the non-bleeding group (OR = 1.609; CI = 1.031-2.509; p = 0.036).

**Conclusion:**

Passive smoking may increase the risk of AVM bleeding in non-smoking women. This increased risk may be related to the inflammatory response, vascular wall damage, hemodynamic disorders, changes in atherosclerosis and changes in gene expression caused by passive smoking.

## RESUMO

**Antecedentes:**

O tabagismo tem sido considerado fator de risco para doenças cardiovasculares, câncer, depressão e outras doenças em relatos anteriores, e o tabagismo ativo é considerado fator de risco para acidente vascular cerebral hemorrágico. Além disso, um estudo retrospectivo mostrou que os fumantes do sexo masculino apresentavam risco aumentado de sangramento por malformação arteriovenosa (MAV), em comparação com os não fumantes. No entanto, o efeito do tabagismo passivo na ruptura da MAV cerebral em mulheres não fumantes não foi abordado. Objetivo: Este estudo teve como objetivo avaliar o impacto da exposição ao tabaco no risco de sangramento de MAV em mulheres não fumantes.

**Métodos:**

Foram incluídas 393 mulheres não fumantes diagnosticadas com MAV. Elas foram divididas em um grupo com sangramento (205 mulheres) e um grupo sem sangramento (188 mulheres). Realizamos análise univariada e multivariada nesses dois grupos. Na análise univariada, foram analisados ​​os fatores de risco que podem estar relacionados ao sangramento de MAV. Na análise multivariada, a relação entre tabagismo passivo e ruptura de MAV foi analisada por meio da correção de fatores de confusão.

**Resultados:**

A análise multivariada mostrou que a proporção de tabagismo passivo foi estatisticamente diferente entre o grupo com sangramento e o grupo sem sangramento (OR = 1,609; IC = 1,031-2,509; p = 0,036).

**Conclusão:**

O tabagismo passivo pode aumentar o risco de sangramento de MAV em mulheres não fumantes. Esse risco aumentado pode estar relacionado à resposta inflamatória, danos na parede vascular, distúrbios hemodinâmicos, alterações na aterosclerose e alterações na expressão gênica causadas pelo tabagismo passivo.

## INTRODUCTION

Tobacco exposure is a healthcare challenge around the world. A survey of adults conducted in 2010 showed that the smoking rates among Chinese men and women were 52.9% and 2.4%, respectively^1^. This means that a large number of non-smoking women may be affected by secondhand smoke. In a 2012 study, the proportion of women who experienced passive smoking was as high as 60.6%^2^. 

The high incidence of passive smoking has always been a social issue that many people are concerned about. It may bring various adverse health problems, which will increase the national medical burden and economic pressure.

Smoking has been considered to be a risk factor for cardiovascular disease, cancer, depression and other diseases in previous reports^3^ ^-^ ^7^, and active smoking is considered to be a risk factor for hemorrhagic stroke^8^. In addition, a retrospective study showed that male smokers were at increased risk of bleeding from arteriovenous malformation (AVM), compared with non-smokers^9^. However, the effect of passive smoking on rupturing of cerebral AVM in non-smoking women has not been addressed. Hence, we designed this study with the aim of assessing the impact of tobacco exposure on AVM bleeding risk in non-smoking women.

## METHODS

### Patient selection

This retrospective analysis study was approved by the Ethics Committee of Beijing Tiantan Hospital and Beijing Jingmei Group General Hospital. Data on patients diagnosed with AVM who were admitted to the Beijing Tiantan Hospital and Beijing Jingmei Group General Hospital between August 2015 and April 2018 were collected. Most of the patients were admitted to the hospital because of symptoms such as headache, nausea, vomiting or epilepsy. A small number of people were also admitted to the hospital because of the presence of AVM, observed during the physical examination. All the cases of AVM were confirmed by means of cerebral angiography. 

The inclusion criteria were as follows: non-smoking women, diagnosed with AVM during hospitalization. The exclusion criteria included the following: (1) the patient's medical records were incomplete or we could not obtain follow-up information, such as lack of digital subtraction angiography (DSA) and computed tomography (CT) data; (2) interventional embolization, surgical resection or radiation treatment were performed before admission; and (3) the patient had one of the following types of malformation: Galen's venous malformation, dural arteriovenous fistula, hereditary hemorrhagic capillary dilatation, cavernous hemangioma, carotid cavernous sinus fistula or pial arteriovenous fistulas. Whether there was bleeding or not was judged through the results from CT/MRI or lumbar puncture, and these examinations were usually completed after admission.

### Data collection and definition of some indicators

In line with reports in the literature, the following indicators were included in our statistical table: passive smoking, age, diabetes, hypertension, ischemic heart disease, family history of stroke, size of AVM, location of AVM, association with blood flow-related intracranial aneurysms (IAs), number of drainage veins, number of feeding arteries, direction of venous drainage, stenoses of drainage veins and dilation of drainage veins.

In this study, non-smoking was defined as having smoked no more than 100 cigarettes in a lifetime. Passive smoking was defined as being exposed to tobacco for at least one hour a day for at least six months before admission. The size of the AVM was divided into three types: small (≤ 3 cm), medium (3-6 cm) or large (≥ 6 cm). With regard to AVM location, cortical referred to the gray matter of the parietal lobe, occipital lobe, frontal lobe and temporal lobe; deep referred to the deep part of the cerebral hemisphere; and infratentorial referred to the cerebellum and brainstem. There were three types of blood flow-related intracranial aneurysms (IAs): proximal aneurysm, distal aneurysm and intra-nest aneurysm. Stenosis and dilation of drainage veins were judged based on whether the diameter of the drainage vein had significantly increased or decreased, compared with normal drainage veins.

The above demographic information was obtained by reviewing medical cases. The vascular architecture characteristics of the AVM were evaluated by two neurointerventional physicians. If there was any dispute about the assessment of the vascular architecture, the evaluation task would be assigned to a third neurointerventional physician. Occurrences of passive smoking were ascertained through a telephone questionnaire survey, using questions such as: “Were there smokers around you every day?” and “How long was each exposure to tobacco?”

### Statistical analysis

We performed univariate and multivariate analyses on the data that had been collected. In univariate analyses, independent-sample t tests were used for continuous variables, and categorical variables were tested using chi-square tests. Variables that were statistically different in univariate analyses were included in the multivariate analysis model. In multivariate analysis, logistic binary regression analysis (LR) was used. For both univariate and multivariate analyses, P-values < 0.05 were considered statistically significant.

## RESULTS

### General characteristics of patients in this study

A total of 393 patients were included in this study. Respectively, 205 were in the bleeding group and 188 were in the non-bleeding group (Table 1). The mean ages of the two groups were 28.6 ± 11.9 and 28.5 ± 12.5 years. The proportions of passive smoking in the two groups were 39.0% and 26.6%.

**Table 1.** Basic characteristics of 393 non-smoking women with cerebral arteriovenous malformations.

Characteristics		Total (393 patients)	Bleeding (205 patients)	Non-bleeding (188 patients)
Age, mean (SD)		28.5 (12.2)	28.6 (11.9)	28.5 (12.5)
Diabetes		16 (4.1%)	9 (4.4%)	7 (3.7%)
Hypertension		50 (12.7%)	25 (12.2%)	25 (13.3%)
Ischemic heart disease		13 (3.3%)	7 (3.4%)	6 (3.2%)
Family history of stroke		34 (8.7%)	17 (8.3%)	17 (9.0%)
Size	Small	160 (40.7%)	100 (48.8%)	60 (31.9%)
	Medium	172 (43.8%)	78 (38.2%)	94 (50.0%)
	Large	61 (15.5%)	27 (13.2%)	34 (18.1%)
Location	Cortical	300 (76.3%)	145 (70.7%)	155 (82.4%)
	Deep	55 (14.0%)	33 (16.1%)	22 (11.7%)
	Infratentorial	38 (9.7%)	27 (13.2%)	11 (5.9%)
Association with blood flow-related IAs		85 (21.6%)	56 (27.3%)	29 (15.4%)
Number of drainage veins (≤ 2)		254 (64.6%)	135 (65.9%)	119 (63.3%)
Number of feeding arteries (≤ 2)		303 (77.1%)	163 (79.5%)	140 (74.5%)
Venous drainage	Superficial	213 (54.2%)	104 (50.7%)	109 (58.0%)
	Deep	96 (24.4%)	47 (22.9%)	49 (26.1%)
	Deep and superficial	84 (21.4%)	54 (26.3%)	30 (16.0%)
Stenoses of drainage veins		27 (6.9%)	10 (4.9%)	17 (9.0%)
Dilation of drainage veins		120 (30.5%)	54 (26.3%)	66 (35.1%)
Passive smoking		130 (33.1%)	80 (39.0%)	50 (26.6%)

IAs: intracranial aneurysms; SD: standard deviation.

### Univariate and multivariate analysis results

Univariate analysis revealed that the risk of AVM bleeding was associated with passive smoking (p = 0.009), size (p = 0.003), location (p = 0.014), IAs (p = 0.004) and direction of venous drainage (p = 0.043). Multivariate analysis showed that the proportion of passive smoking was statistically different between the bleeding group and the non-bleeding group (OR = 1.609; CI = 1.031-2.509; p = 0.036). In addition, multivariate analysis results showed that size (medium vs small: p = 0.001; large vs small: p = 0.023), location (infratentorial vs cortical: p = 0.009) and association with IAs (p = 0.006) were statistically different between the two groups (Table 2).

**Table 2.** Univariate and multivariate regression analysis results relating to passive smoking and AVM bleeding among non-smoking women with AVM.

Characteristics		Univariate	Multivariate		
		p-value	OR (95% CI)	p-value
Age		0.936		
Diabetes		0.738		
Hypertension		0.743		
Ischemic heart disease		0.902		
Family history of stroke		0.792		
Size	Small	0.003	Reference	
	Medium		0.470 (0.298-0.741)	0.001
	Large		0.490 (0.265-0.906)	0.023
Location	Cortical	0.014	Reference	
	Deep		1.609 (0.880-2.942)	0.122
	Infratentorial		2.795 (1.299-6.014)	0.009
Associated with blood flow-related IAs		0.004	2.072 (1.232-3.487)	0.006
Number of drainage veins (≤ 2)		0.597		
Number of feeding arteries (≤ 2)		0.235		
Venous drainage	Superficial	0.043		
	Deep			
	Deep and superficial			
Stenoses of drainage veins		0.103		
Dilation of drainage vein		0.059		
Passive smoking		0.009	1.609 (1.031-2.509)	0.036

IAs: intracranial aneurysms; CI: confidence interval.

## DISCUSSION

### Passive smoking may increase the risk of AVM bleeding in non-smoking women

Exposure to secondhand smoke for various reasons is called passive smoking. The smoke generated from smoking cigarettes contains harmful substances, such as nicotine. Passive smoking mainly takes place at home or at work. In China, the majority of passive smokers are women. A study conducted by Yao et al. showed that Chinese women's medical expenses due to the effects of secondhand smoke were as high as $900 million^10^. Passive smoking increases not only the cost of healthcare but also the risk of several other diseases, such as lung cancer, coronary heart disease, adult chronic respiratory disease and childhood asthma^11^. Smoking has also been demonstrated to be a risk factor for some subtypes of hemorrhagic stroke^12^. A retrospective analysis conducted by Ming Lv also showed that male smokers were at increased risk of AVM rupture^9^.

In our study, the rate of passive smoking in the bleeding group was about 39.0%, and in the non-bleeding group it was 26.6%. Univariate analysis showed that there was a statistical difference in the proportions of passive smoking between the two groups (P = 0.009). After excluding the influence of confounding factors, the results from multivariate analysis also showed that there was a statistical difference in the proportions of passive smoking between the two groups (P = 0.036). The proportion of passive smoking in the bleeding group was higher than that in the non-bleeding group. This suggests that passive smoking may be a risk factor for bleeding in women with AVM.

In addition, the multivariate analysis result showed that the following factors may also be related to the risk of AVM bleeding in the non-smoking female population: size, location and association with IAs. These factors were not the focus of this study and will not be discussed here. In our previous studies, it was found that there may be a correlation between smoking and AVM bleeding risk among males^9^. 

The uniqueness of the present study is that, for the first time, the relationship between the exposure factors for passive smoking and the risk of AVM bleeding in non-smoking females was analyzed. As is well known, the proportion of Chinese women who smoke is relatively small, but they face the threat of smoke exposure brought by men when they smoke. In this study, a quantitative method was adopted for the factor of passive smoking, and the method of multivariate analysis was used to minimize the occurrence of bias.

### Possible mechanism

**Figure 1.**
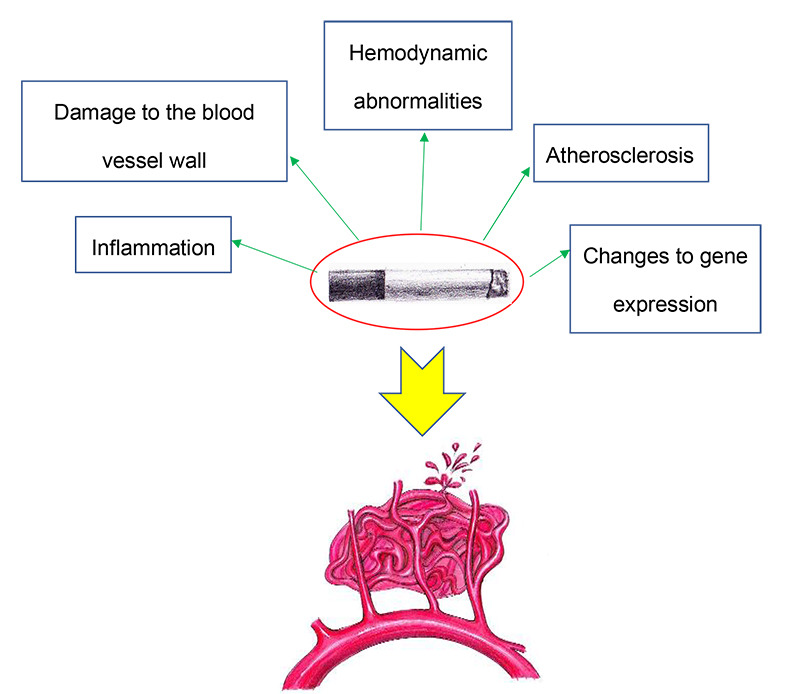
Possible mechanisms for AVM bleeding caused by passive smoking among non-smoking women.

This study mainly focused on the association between passive smoking and AVM bleeding in non-smoking women. Therefore, we will address the possible mechanisms through which passive smoking may increase the risk of AVM bleeding. These may include the following aspects (Figure 1):

(1) Inflammation: Tobacco-burning smoke can recruit inflammatory cells via increased fibrinogen^13^. Inflammatory cells in AVM can produce substances that cause inflammation or instability of blood vessel walls, such as signal transduction molecules or molecules related to enzyme activity. Among these molecules, metalloproteinase 9 and peroxidase can break down collagen, fibrin, laminin and other substances on the basement membrane of blood vessel^14^ ^-^ ^18^. 

(2) Damage to the blood vessel wall: Tobacco smoke can cause damage to blood vessel walls. The endothelial cells of the blood vessel wall can be affected by aromatic compounds, and the functioning of these cells is weakened. Accordingly, the production of nitric oxide will be reduced, and endothelium-related diastolic functioning will gradually decrease^19^. Vascular remodeling will occur after vascular wall injury and this can lead to bleeding events.

(3) Hemodynamic abnormalities: Epidemiological studies of the normal population have suggested that smoking could cause vasospasm^20^. Simultaneously, carbon monoxide in smoke can inhibit combination of hemoglobin and oxygen molecules, such that red blood cells are compensated and blood viscosity is increased^21^. Furthermore, compared with non-smokers, the average blood pressure of smokers also increases^22^.

(4) Atherosclerosis: During the smoking process, exposure of the matrix components of the vascular wall may occur, in which collagen activates adhesion and aggregation of platelets on the endothelium. The aggregated platelets further secrete platelet-derived growth factor (PDGF). Mononuclear cells gradually migrate to the intimal layer and differentiate into macrophages, which accelerates the process of atherosclerosis^19^. This makes the vessel wall more brittle and increases the risk of bleeding. 

(5) Changes to gene expression: Gene chip technology was used by Miao et al. and they found that smoking can induce multiple gene expressions, such as ZFP36, PTGS2, NFKBIZ and TNFAIP3^23^. Among these genes, PTGS2 may increase the risk of AVM bleeding through the NF-κB signaling pathway and the inflammatory response pathway. NFKBIZ can also mediate inflammatory responses through interaction between ankyrin and NF-κB protein^24^. 

### Limitations

Firstly, this study was a retrospective study with limited statistical power, rather than a prospective cohort study. In addition, the number of patients enrolled in this study was not enough, and statistical bias was inevitable. Furthermore, the AVM patients included were inpatients and no outpatients were included. There may have been some selection bias. Moreover, the survey of passive smoking was conducted through telephone interviews. There was also a lack of investigation of the relationship between dose and response. Lastly, the population of this study was from China. In contrast, among populations in Europe, the United States and other countries, women's exposure to passive smoking is not the same as in China. Therefore, whether the results from this study are universal remains uncertain.

In conclusion, passive smoking may increase the risk of AVM bleeding in non-smoking women. This increased risk may be related to the inflammatory response, vascular wall damage, hemodynamic disorders, changes to atherosclerosis and changes to gene expression caused by passive smoking.
